# Impact of faculty development programme on self-efficacy, competency and attitude towards medical education in Bhutan: a mixed-methods study

**DOI:** 10.1186/s12909-019-1904-4

**Published:** 2019-12-21

**Authors:** Karma Tenzin, Thinley Dorji, Tshering Choeda, Krit Pongpirul

**Affiliations:** 1 Faculty of Postgraduate Medicine, Khesar Gyalpo University of Medical Sciences of Bhutan, Thimphu, Bhutan; 2 Jigme Dorji Wangchuck National Referral Hospital, Thimphu, Bhutan; 3 Kidu Mobile Medical Unit, His Majesty’s People’s Project, Thimphu, Bhutan; 4 Department of Preventive and Social Medicine, Faculty of Medicine, Chulalongkorn University, Bangkok, Thailand; 5 Department of International Health, Johns Hopkins Bloomberg School of Public Health, Baltimore, MD USA

**Keywords:** Core competency, Health human resource, Health professional education, Residency training

## Abstract

**Background:**

Soon after Bhutan’s first medical university was established in 2012, Faculty Development Programmes (FDPs) were adopted for efficient delivery of postgraduate medical curriculum. Medical education was an additional responsibility for the clinicians who already had multi-dimensional roles in the healthcare system where there is acute shortage of healthcare professionals. We studied the impact of FDPs on postgraduate medical education in Bhutan.

**Methods:**

This was a mixed-methods study with a quantitative (cohort study – quasi-experimental with 18 participants) and concurrent explanatory qualitative component (focused group discussion (FGD) with 11 teaching faculty members). The 18 participants were given a structured FDP designed by the University. The FGD assessed teacher self-efficacy and competency using standard tools before and after the FDP. Thematic analysis of the FGD explored the impact of FDPs in the delivery of postgraduate residency programmes.

**Results:**

There were significant increase in the teacher self-efficacy (31 vs 34, *p* = 0.009) and competency scores (56 vs 64, *p* = 0.011). There were significant improvements in self-efficacy in the domain of the teaching relevant subject contents and developing creative ways to cope with system constraints. In teaching-learning assessments, there was a significant appreciation of the effectiveness of lectures and tutorials and the use of essay questions. The FGD demonstrated the acceptance of FDPs and its importance in quality improvement of postgraduate medical education, professional development of teachers and improvement of their communication skills. The teachers have now migrated from the conventional methods of teaching to workplace-based teaching and assessment. The FDPs also resulted in review and revision of postgraduate medical curriculum soon after the first batch graduated in 2018. Lack of adequate support from relevant stakeholders and lack of a medical education centre in the University were seen as major challenges.

**Conclusions:**

The FDPs have brought tangible professionalization of postgraduate medical education at an early stage of the medical university. There is a need for continued efforts to strengthen, sustain and consolidate the gains made thus far.

## Background

Faculty Development Programmes (FDPs) are an important aspect of medical education and in efficient delivery of medical curriculum [1, 2]. Over the last decade, there have been major changes in the context in which FDPs were delivered around the globe and in South East Asia [1, 3]. FDPs involve activities that improve the knowledge, skills, and behaviours of health professionals as teachers and educators, as leaders and managers, and as researchers and scholars, in both individual and group settings [4].

FDPs are central to delivering medical education that is responsive to changes in the health care system, evolving social expectations of the patients and the shift in medical learning to workplace-based approaches [5–8]. With shortage of health human resources and the increased demand for medical education [2, 3], regulatory medical councils in the South East Asia region prescribe FDPs to enhance the quality of medical education [3, 4, 6]. However, for many newly established medical colleges, FDPs are a means to induct faculty members into continuing professional development and increase their capacity to get involved in leadership and management in the university, hospital and the community; and in research and scholarship [2, 4, 9].

In Bhutan, it was only in 2018 that the first batch of eight postgraduate doctors that were trained within the country, graduated from the Khesar Gyalpo University of Medical Sciences of Bhutan, Thimphu [10]. This was the result of the establishment of the University of Medical Sciences of Bhutan in 2012 and the Faculty of Postgraduate Medicine in 2014 [11–13]. For residency training, the Faculty of Postgraduate Medicine has eight faculty members for basic sciences subjects and 90 core and adjunct faculty members in clinical subjects from three teaching hospitals: Jigme Dorji Wangchuck National Referral Hospital (JDWNR Hospital), Thimphu; Central Regional Referral Hospital, Gelegphu; and Eastern Regional Referral Hospital, Monggar with a combined capacity of 680 beds [10].

When the Faculty of Postgraduate Medicine began, the existing clinicians at the JDWNR Hospital, Thimphu took up residency teaching and FDPs to add to their already multi-dimensional role [2, 14, 15]. FDPs were therefore adopted as a short-term goal to enhance teaching effectiveness, implement the postgraduate curriculum and to develop medical education that is specific to the context of Bhutan [16]. With no medical educationists in the country, the University sought experts from other institutions (India, Nepal, Thailand and Germany) to design and implement a series of FDP workshops and training [10, 12].

Given that almost all of the faculty members were trained in the conventional system of postgraduate education, these programmes marked a significant shift in the adoption of modern tenets of medical education. In this study, we attempted to document the self-efficacy and competencies of the teachers, their utilisation of various teaching-learning and assessment methods and the impact of FDPs on various aspects of postgraduate teaching-learning in Bhutan.

## Methods

### Study design

This was a mixed-methods study with a quantitative (cohort study – quasi-experimental), and concurrent explanatory qualitative component (focused group discussion with teaching faculty members) reported using the COREQ guidelines [17, 18].

### FDP interventions

The FDP interventions were designed by the Faculty of Postgraduate Medicine based on need assessment. The programme contents were reviewed and approved by medical education experts from India, Nepal, Thailand and Germany [10, 12]. Parts of the workshop were conducted in Bhutan and parts in medical colleges in India, Thailand and Germany. All the FDPs conducted in Bhutan had a mix of relevant international experts. The FDPs were provided within the framework of the development of medical education, professional development of teachers and quality improvement of postgraduate medical education [7, 10, 19]. The themes included adult learning and foundation of education; education objectives and lesson planning; giving effective lectures; teacher’s role in mentoring; microteaching; teaching replay and feedback; concept and tools of evaluation, construction of multiple choice questions and objective structured clinical examinations; workplace-based assessments, academic leadership and educational research [12].

The faculty members were given 12 months’ time to develop expertise through experience, observation, reflection, peer coaching, learner feedback, online learning, and workplace-based learning [1]. The subsequent FDPs transitioned to adopt the expanded model on medical education and residency teaching within the context of workplace-based learning and assessment [1, 7, 12, 20].

### Quantitative component

#### Study site

This quantitative component was conducted with faculty members from the Faculties of Postgraduate Medicine, Nursing and Public Health, and Traditional Medicine, Khesar Gyalpo University of Medical Sciences of Bhutan; and adjunct faculty members from the JDWNR Hospital, Thimphu.

#### Study participants

The study involved faculty members who underwent the FDP programmes organized by the Faculty of Postgraduate Medicine, Thimphu in 2017 and 2018 [10]. For this cycle of FDP, based on the availability of funds, only those faculty members who were not trained before or exposed to FDPs were selected.

#### Data collection method

The participants filled the study questionnaire before the initiation of any of the FDP workshops in 2017 and filled the same questionnaire after the FDP cycle at the end of 2018. The study questionnaire consisted of teacher’s self-efficiency test, assessment of teaching competencies and a self-designed rating scale to assess their attitudes towards teaching and evaluation methods.

The teacher self-efficacy test is a 10-item scale used to measure self-efficacy in teaching in relation to four areas: job accomplishment, skill development, social interactions with students and colleagues, and coping with job stress [21]. It is a self-reporting four-point Likert scale. Scores range from 10 to 40 with higher scores representing higher teacher self-efficacy. Assessment of teaching competencies was done in seven domains: communication skills, engaging and supporting all students, creating and maintaining an effective environment, understanding and organizing subject matter, planning instruction and designing learning experiences, assessing student learning, and developing as a professional educator. It is also a self-reported Likert scale. Scores range from 20 to 80 with higher scores representing higher competency in teaching. The attitudes of the faculty members on various teaching methods, and media used for teaching and assessment were collected using a four-point rating scale: not effective, somewhat effective, effective and very effective. Informed consent waiver was granted by the ethics board for this component of the study.

#### Data entry and analysis

Data were double entered and validated in January 2019 using EpiData 3.1 (EpiData Association, Odense, Denmark) and analysed using STATA Version 13.1 (StataCorp. 2013. *Stata Statistical Software: Release 13*. College Station, TX: StataCorp LP). Descriptive statistics are presented as frequencies and percentages. Scores before and after the training were compared using t-tests. Changes around the themes of self-efficacy, teaching competency, and attitudes on the effectiveness of teaching methods, and teaching media and assessment methods before and after the FDPs were tested using chi-square test. Attitude statements that were scored as “not effective” and “somewhat effective” are presented as “not effective” and those scored as “effective” and “very effective” are presented as “effective.” *P* value < 0.05 were considered significant.

### Qualitative component

#### Study site

The qualitative component was conducted with faculty members from the Faculty of Postgraduate Medicine, Khesar Gyalpo University of Medical Sciences of Bhutan; and adjunct faculty members from the JDWNR Hospital, Thimphu.

#### Study participants

A total of 11 faculty members participated in the FGD: six were males. The departments represented were: Anaesthesiology, Biochemistry, Ear-Nose-Throat (ENT), Emergency Medicine, General Practice, Obstetrics and Gynaecology, Ophthalmology, Orthopaedic Surgery, Paediatrics, and Psychiatry. The participants were the coordinators of the residency programme in their respective departments. Seven participants have had residency programme in their departments for 4 years, two have had for 2 years and two did not have residency programme in their departments at the time of the interview.

#### Data collection and analysis

The FGD was conducted in English using an interview guide (Additional file 1) in October 2018 at an FDP retreat in Paro district. The FGD was facilitated by KT (MD, Fellowship in Medical Education with experience in qualitative research) with the participants seated in a circle. The participants knew the facilitator as he was an assistant professor in physiology at the Faculty of Postgraduate Medicine. The interview began after the participants were given an overview of the exercise and how this FGD would contribute to the evaluation and development of FDP in the University. The participants were given a printed copy of the interview guide. TC observed the FGD but was seated outside the circle and did not contribute to the discussion. The participants were given the information that TC would observe the FGD and help in the transcription of the voice recording. Data collection was continued until saturation was achieved, i.e. until participants provided no additional information. The principal investigator took informed written consent for participation in the FGD and voice recording.

The voice recording was transcribed by TD within 2 days of the FGD, and TC clarified three instances of lack of voice clarity in the recording. The FGD had lasted 75 minutes. KT, TD and KP manually identified themes that emerged from the FGD and identified quotations that illustrate the themes. The quotations are given against participant number as (P1, P2 … P11). Inconsistencies in the thematic analyses were resolved through discussion among the investigators.

### Ethics approval

Ethics review was exempted by the Research Ethics Board of Health, Ministry of Health, Bhutan given the non-biomedical nature of the study; however, informed written consent was taken for the qualitative component. The study was conducted after obtaining permission from the Faculty of Postgraduate Medicine. Only anonymised data is presented in this and all identifiers related to the quotations are removed.

## Results

### Quantitative component

There were 18 faculty members who underwent a series of FDPs in 2017–2018: 12 (67%) from the Faculty of Postgraduate Medicine, 4 (22%) from the Faculty of Nursing and Public Health, and 2 (11%) from the Faculty of Traditional Medicine. The median year of teaching experience was 1 year (IQR 0.4, 3).

The mean score of teacher self-efficacy increased from 31 (±4) out of 40 to 34 (±4), *p* = 0.009, (Table 1). There were significant improvements in teaching relevant subject contents (*p* = 0.016) and developing creative ways to cope with system constraints (*p* = 0.001). The mean score of teaching competency increased from 56 (±2) out of 80 to 64 (±3), *p* = 0.011 (Table 2). There were significant improvements in the appreciation of the effectiveness of lectures (*p* = 023) and tutorials (*p* = 0.020) for teaching; use of Microsoft Word (*p* = 0.004) for writing case history or case reports; and the use of essay questions (*p* = 0.032) in assessments. Their attitude towards methods of teaching, medium of teaching and methods of evaluation are shown in Table 3.

**Table 1:** Assessment of teacher self-efficacy before and after participation in a series of faculty development programmes provided by the Khesar Gyalpo University of Medical Sciences of Bhutan, 2017–2018

	Before FDPs				After FDPs				Chi-square *p*-value
	Not efficacious		Efficacious		Not efficacious		Efficacious		
	n	(%)	n	(%)	n	(%)	n	(%)	
Teach all relevant subject content to even the most difficult students	5	(28)	13	(72)	2	(11)	16	(89)	**0.016**
Maintain a positive relationship with students even when tensions arise	1	(6)	17	(94)	0	(0)	18	(100)	–
Reach even the most difficult students	3	(17)	15	(83)	1	(6)	17	(94)	0.645
Become more capable of helping to address my students’ needs	0	(0)	18	(100)	0	(0)	18	(100)	–
Maintain my composure and continue to teach well even when I am disturbed	0	(0)	18	(100)	0	(0)	18	(100)	–
Responsive to my students’ needs even if I am having a bad day	1	(6)	17	(94)	0	(0)	18	(100)	–
Exert a positive influence on both the personal and academic development of my students	1	(6)	17	(94)	0	(0)	18	(100)	–
Develop creative ways to cope with system constraints and continue to teach well	3	(17)	15	(83)	2	(11)	16	(89)	**0.001**
Motivate my students to participate in innovative projects	2	(11)	16	(89)	0	(0)	18	(100)	–
Carry out innovative projects even when I am opposed by sceptical colleagues	4	(22)	14	(78)	0	(0)	18	(100)	–

Boldface entries are statistically significant

**Table 2:** Assessment of teaching competency before and after participation in a series of faculty development programmes provided by the Khesar Gyalpo University of Medical Sciences of Bhutan, 2017–2018

	Before FDPs				After FDPs				Chi-square *p*-value
	Not competent		Competent		Not competent		Competent		
	n	(%)	n	(%)	n	(%)	n	(%)	
Communicating effectively with students’ level of knowledge	0	(0)	18	(100)	2	(11)	16	(89)	–
Communicating effectively to bring about behavioural or attitudinal change	5	(28)	13	(72)	2	(11)	16	(89)	0.352
Connecting students’ prior knowledge to the learning goals	3	(17)	15	(83)	1	(6)	17	(94)	0.645
Engaging students in problem solving and critical thinking	5	(29)	12	(71)	1	(6)	17	(94)	0.331
Promoting self-directed reflective learning	5	(29)	12	(71)	2	(11)	16	(89)	0.331
Creating physical environment that engages the students	7	(41)	10	(59)	1	(6)	17	(94)	0.388
Promoting individual as well as group responsibility	2	(11)	16	(89)	1	(6)	17	(94)	0.716
I have adequate subject knowledge	0	(0)	18	(100)	2	(11)	16	(89)	–
Organising teaching lessons in a sequence that supports students’ understanding of the subject matter	5	(28)	13	(72)	1	(6)	17	(94)	0.523
Use resources, materials and technologies to make subject matter more accessible	8	(44)	10	(56)	2	(11)	16	(89)	0.094
Planning lessons or modifying my instructional plans depending on students’ learning needs	11	(61)	7	(39)	1	(6)	17	(94)	0.412
Designing short-term and long-term learning goals to foster students’ learning	8	(44)	10	(56)	1	(6)	17	(94)	0.357
Establishing and communicating learning goals to my students	4	(24)	13	(76)	0	(0)	18	(100)	–
Collecting multiple sources of information to assess students’ learning	8	(44)	10	(56)	2	(11)	16	(89)	0.094
Involving students to assess their own learning	9	(50)	9	(50)	1	(6)	17	(94)	0.303
Communicating with students on their progress of learning	3	(18)	14	(82)	2	(11)	16	(89)	0.486
Reflecting on teaching practice and planning professional development	9	(50)	9	(50)	1	(6)	17	(94)	0.303
Establishing professional goals and pursuing opportunities to learn more on medical education	8	(44)	10	(56)	2	(11)	16	(89)	0.867
Working with colleagues to improve teaching practices	4	(22)	14	(78)	3	(17)	15	(83)	0.612
Balancing teaching responsibilities and maintaining motivation	9	(50)	9	(50)	2	(11)	16	(89)	1.000

**Table 3:** Attitude towards methods of teaching and evaluation before and after participation in a series of faculty development programmes provided by the Khesar Gyalpo University of Medical Sciences of Bhutan, 2017–2018

	Before FDPs				After FDPs				Chi-square *p*-value
	Not effective		Effective		Not effective		Effective		
	n	(%)	n	(%)	n	(%)	n	(%)	
Methods of teaching									
Lecture	7	(41)	10	(59)	4	(22)	14	(78)	**0.023**
Tutorial	5	(29)	12	(71)	2	(11)	16	(89)	**0.020**
Group discussion	2	(12)	15	(88)	0	(0)	18	(100)	–
Problem based learning/ case scenario	2	(12)	15	(88)	1	(6)	16	(94)	0.790
Seminar/presentation	5	(29)	12	(71)	1	(6)	17	(94)	0.506
Role play	2	(12)	14	(88)	1	(6)	17	(94)	0.696
Brain storming	3	(18)	14	(82)	1	(6)	17	(94)	0.633
Medium of teaching									
Black-board teaching	12	(71)	5	(29)	3	(17)	15	(83)	0.218
Overhead projector	7	(41)	10	(59)	12	(71)	5	(29)	0.197
PowerPoint presentation	3	(18)	14	(82)	3	(18)	14	(82)	0.226
Microsoft Word	6	(37)	10	(63)	6	(35)	11	(65)	**0.004**
Online videos	2	(12)	15	(88)	4	(22)	14	(78)	0.486
Websites	5	(29)	12	(71)	3	(17)	15	(83)	0.496
Methods of evaluation									
Multiple choice questions	4	(25)	12	(75)	1	(6)	17	(94)	0.074
Fill in blanks	7	(47)	8	(53)	7	(39)	11	(61)	0.072
Labelling of diagrams	7	(50)	7	(50)	5	(28)	13	(72)	0.577
Match the items	12	(80)	3	(20)	10	(56)	8	(44)	0.171
Short answer questions	3	(20)	12	(80)	0	(0)	18	(100)	–
Essay questions	5	(33)	10	(67)	2	(11)	16	(89)	**0.032**
Short notes	6	(43)	8	(57)	3	(17)	15	(83)	0.078
Assignments	3	(27)	8	(73)	1	(7)	13	(93)	–
Viva voce	5	(38)	8	(62)	4	(22)	14	(78)	0.715
Objective structured practical examination	1	(7)	14	(93)	1	(6)	17	(94)	0.782
Laboratory practical	3	(20)	12	(80)	4	(22)	14	(78)	0.519
Bedside examination	2	(14)	12	(86)	1	(6)	15	(94)	–

Boldface entries are statistically significant

### Qualitative

**Fig. 1:**
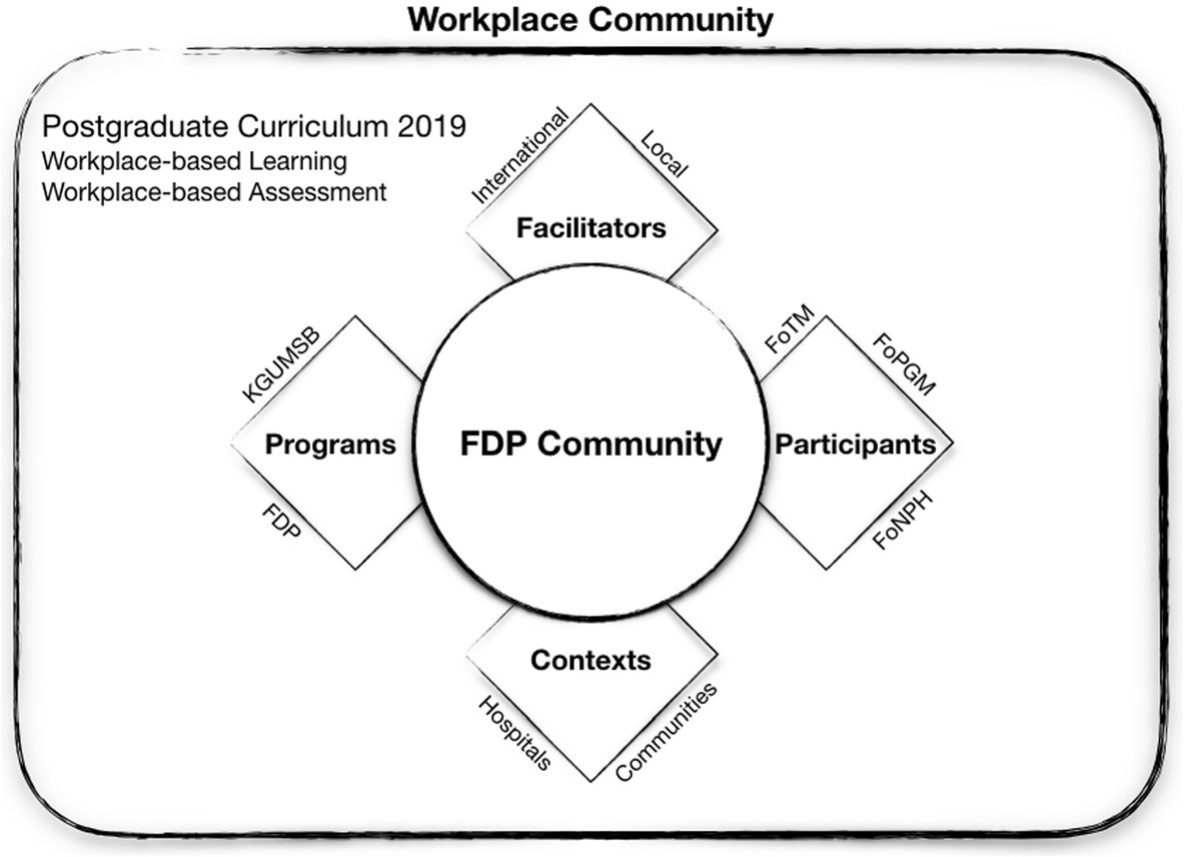
The expanded model for faculty development programme with the budding FDP community and workplace-based teaching-learning environment at the Khesar Gyalpo University of Medical Sciences of Bhutan, 2019. FDP: faculty development programme, FoNPH: Faculty of Nursing and Public Health, FoPGM: Faculty of Postgraduate Medicine, FoTM: Faculty of Traditional Medicine, KGUMSB: Khesar Gyalpo University of Medical Sciences of Bhutan

The FGD explored the views of faculty members towards FDPs and their impact in postgraduate teaching at the Faculty of Postgraduate Medicine. The FGD brought contextual examples on the adoption and implementation of themes around teaching self-efficacy, teaching competency and the teaching and assessment methods. An overview of the findings is shown in Fig. 1 using the expanded model of FDPs [7].

### Importance of FDPs

The participants had recognized the importance of FDPs in the context of postgraduate medical education in the country. The importance was expressed within the frameworks of quality improvement of postgraduate education, professional development of the teacher, and workplace-based learning environment with an overall aim of delivering to the improvement of not only the knowledge and skills but also the cognitive domain of the student.

*“I can now add on to the development of the affective domain of the student and contribute to making a better doctor.” – P4*

*“It is equally important for the Faculty of Postgraduate Medicine to train the faculty members from the departments that have started their residency programme after us.” – P11 who had been running residency programme at the department for four years.*

*“We hope to have a new group of doctors, who are not only competent in their clinical skills but compassionate and caring towards their patients.” – P9*

#### Quality improvement

For quality improvement, the FDP activities have helped identify gaps between the current practices in our setting and those in the institutions in the region and in developed countries. The participants also highlighted the changes that were adopted as a result of the FDPs.

*“After the FDPs, I was able to organise our clinical and teaching activities that were efficient for both the patients and the residents.” – P4, postgraduate resident coordinator and head of department*

*“After having had several sessions, I can now make MCQs with good distractor effectiveness.” – P4*

*“After the FDPs, in addition to the MCQs, we now conduct OSPEs in the entrance examination for PG courses.” – P5*

#### Professional development

In the context of professional development as a teacher, the participants highlighted the limitations of the postgraduate training they had received under the conventional system. They acknowledged the potential benefits of exposure of the postgraduate trainees to the principles of medical education as a part of the foundational curriculum at the Faculty of Postgraduate Medicine [12].

*“When the batch of residents who studied medical education as a part of their curriculum graduate, FDP in the Faculty of Postgraduate Medicine is set to go in the right direction.” – P6*

FDPs were recognized as an important aspect of adult learning and the participants called for more active participation from the Faculty of Nursing and Public Health and the Faculty of Traditional Medicine.

*“While Faculty of Postgraduate Medicine has adopted FDPs and new teaching and learning methods, I would like to know the development and implementation of FDPs in the Faculty of Nursing and Public Health and Faculty of Traditional Medicine.” – P11*

### Faculty development programmes

#### Methods of teaching

The workshops introduced the principles of adult learning, competencies of the teacher, understanding and organizing the subject matter, the appropriate methods of assessment and the delivery of the revised postgraduate curriculum. Compared to the times they had undergone their postgraduate training, the participants, who were current faculty members, reported that the major difference now is the easy access to information. In this light, the FDPs have helped the faculty members to adopt online learning and the use of information and technology. However, the faculty members emphasized the role of providing proper guidance in these type scenarios.

*“Nowadays, knowledge is accessible at the tip of your finger. We should guide our residents to the right sources of knowledge and help them become good doctors.” – P6*

*“We share journal articles on recent developments online and run sessions on critical appraisal in the form of group discussions.” – P5*

Guiding residents towards an appropriate source of information and knowledge requires understanding and organizing the subject matter. FDPs have helped them identify learning objectives and improve the efficiency of the delivery of the subject matter.

*“I have been teaching postgraduates before and after the Faculty Development Programme. After the programme, I was able to understand and better organize my teaching materials and lectures.” – P5*

*“The FDP workshops helped identify appropriate teaching materials and parts of the curriculum to be delivered based on the year the residents are in the programme.” – P7*

*“As a professional educator, we need to understand the core subject that the resident needs to know so that we can provide effective guidance.” – P10*

The FDP has also helped the faculty members understand which teaching methods are appropriate for their department. The participants recognised the importance of developing their residents to a life-long adult learner. The participants highlighted how skills such as history taking and examination and performing surgeries and procedures are imparted in their workplace-based environment using the expanded model of FDPs (Fig. 1).

*“We provide hands-on training and guide the residents on performing surgeries step-by-step and case-by-case. We did not have this opportunity during our times elsewhere.” – P10*

*“We have simulated learning sessions related to anaesthesiology skills. We demonstrate the skills and the residents practice on mannequins.” – P6*

Given that it can be performed in routine settings, the use of case-based discussions and bed-side teaching were common across all departments. The participants reported how opportunities were used to provide objective learning sessions to their residents.

*“Bed-side teaching are very useful in clinical settings and we can demonstrate how to perform patient examination.” – P10*

There were contending opinions with regard to the use of PowerPoint presentations. Some departments made frequent use of it in the form of resident presentations and symposia. The Emergency Department shared their experiences of how PowerPoint presentations have been used effectively to deliver information to a diverse group of students. One department shared how faculty presentation is used to teach residents as well as in providing continuing medical education to the other faculty members and technicians.

*“We use PowerPoint presentations for seminar where we teach some core topics, and for case presentation where we have discussion on topics around the case.” – P7*

*“PowerPoint presentations are very helpful in reaching out to wider and diverse audience: residents, interns, medical officers, nurses and emergency responders. Besides, the person making the PowerPoint reads and learns a lot.” – P1, emergency department*

*“Every month, a faculty member gives a lecture on recent advances. Weeks before this session, reading materials such as journal articles are shared online and everyone made to read them.” – P4*

However, some shared their opinion that such conventional didactic teaching was at risk of sharing outdated information and possible limitation of the scope of learning only to the topics covered in those presentations. It was also shared that presentations were predominantly done by the residents and overseas volunteer doctors, and not by the faculty members. In the quantitative analysis, the FDPs did not result in any change in the attitude towards the use of PowerPoint presentations (Table 3).

*“I think better learning materials are available on the internet than PowerPoint presentations.” – P11*

*“We tend to use the same slides to teach many batches over years and oftentimes we miss the recent updates.” – P4*

The FDP has also encouraged the departments to find means of enhancing self-efficacy in teaching-learning. Departments have mobilised resources and expertise to provide relevant and necessary knowledge and skills to their residents.

*“We have initiated inter-departmental case conferences and invite colleagues from other departments such as radiology and pathology to help us with difficult patient cases.” – P6*

#### Methods of assessment

The participants recognised the departure from conventional methods of assessment and the dynamics of assessment in a workplace-based environment. The participants have understood the importance and rationale behind newer assessment methods and welcomed the introduction of workplace-based assessments and 360-degree assessments. However, it was highlighted that almost all of them have graduated through conventional means of assessments that have its own set of advantages.

*“At the end of the day, we want residents who are competent to provide appropriate care to the patients. Through the right methods of assessment, we can make sure that the residents have learned what is required of them.” – P1*

*“We can now assess our students with appropriate methods without bias and prejudice that were associated with the traditional system of assessment.” – P10*

Assessment methods such as direct observation of procedural skills were welcomed as it establishes the legitimacy of the student learning and trustable professional activities under the Bhutan Medical and Health Council regulations and also acts as a certification of their skills as they pass through senior years of residency.

*“We have adopted DOPS (direct observation of procedural skills) where we assess and document the improvement of procedural skills of our residents.” – P6*

However, some students have raised pertinent questions on the adoption of such assessment methods given that the University lacks a medical education centre to monitor and evaluate the effectiveness of such initiatives.

*“The residents questioned whether these assessment methods are standard and if these were adopted after careful thinking of its suitability in our setting.” – P9*

Therefore, with a lack of experience in having implemented the new assessment methods, the participants also emphasized the need for strengthening of conventional forms of assessment.

*“We should not overlook the role of conventional forms of assessment that are well tested, and that we are much familiar with. I believe in the combination of workplace-based assessment with conventional forms so that the downside of one method is offset by the other.” – P1*

*“We have retained the assessment methods that were good in the old curriculum. We have adopted several new methods in the revised curriculum. We would like to see how it complements each other.” – P5*

#### Communication skills

The FDPs have also brought conscious changes in the improvement of communication with patients and the doctor-patient relationship. The participants recognized the influence of the socio-cultural context of communication with colleagues, residents, and patients.

*“To be an effective teacher, I have realised that it requires you to create effective environment for learning and giving clear instructions to the students.” – P11*

*“With these workshops, I am now able to communicate effectively not only with my residents but also with my colleagues.” – P9*

*“I am now able to listen actively to them [residents] as well as teach them the subject matter.” – P3*

### Context of residency teaching at JDWNR Hospital

The majority of the residency training programme happens at the JDWNR Hospital, Thimphu. The hospital being the apex referral point in the country, offers a unique set of challenges and opportunities in postgraduate education. The participants discussed the efforts taken by their departments in creating effective learning environment for their residents. The departments have created spaces such as lecture room, skills lab and resting lounges for their residents.

*“We converted a room in the hospital into lecture room where we can have teaching-learning activities without disturbance, away from the routine work in the ward.” – P9*

*“We have a skills lab that provides training and certification BLS and ACLS courses. We welcome participants from the whole country and our residents get to attend and facilitate these courses.” – P1, emergency department*

However, the participants also shared the challenges they face in finding a balance between patient care and resident teaching.

*“Sometimes the Emergency Department can become too crowded and chaotic for resident learning. Our bedside teaching sessions frequently get disrupted. Sometimes we are unable to complete bedside teaching because we have to attend to patients.” – P1, emergency department*

*“We are short-staffed and we have patients waiting. We can devote very little time for resident teaching.” – P11*

The participants also expressed their frustration on the lack of adequate support for resident teaching from hospital administrators and policymakers who control the hospital. The participants emphasized the continued state of non-recognition of the residents’ contribution in service delivery in the hospital.

*“The hospital administrators need to acknowledge how much the consultants and residents give back, through the residency programme, to the hospital and the patients.” – P11*

### Facilitators for FDP

The FDPs were conducted with experts from outside the country. While this allows for the import of new information and techniques, the disadvantage is the lack of continued contact with the experts for mentoring and coaching in a step-by-step process. With a lot of demand for facilitation and support for FDP activities at the department level, the Faculty of Postgraduate Medicine has initiated a process to establish a medical education unit.

*“In the future, we must train all of our faculty members in FDP and develop our own experts in medical education and design a postgraduate programme that is suited to our local context.” – P6*

### Workplace-based learning and assessment

The participants also noted a shift in the paradigm of postgraduate education to workplace-based learning with the introduction of FDPs and the revised curriculum in 2018. Workplace-based learning and assessment seeks active participation in the nurturing of an all-around resident: affective domain, cognitive domain, and skills. The 360 degrees assessment involves a formative assessment of a resident by all members of the patient care team in the hospital.

*“Workplace-based assessment and feedback is important because they will be practicing in a similar setting after they graduate.” – P10*

### Challenges for the continuation of FDPs

While FDPs have benefitted those departments whose faculty members were trained, some challenges are foreseen in terms of sustainability of the FDPs.

*“When we started the FDPs, it was difficult to get adequate funds and support.” – P11*

*“Even after four years of teaching residents, what I had prepared as an OSCE were not OSCEs in true sense. So, there is a need for further support.” – P9*

*“These concepts are new to all of us. We would need support until we are confident to implement all of them effectively.” – P6*

## Discussion

The FDP has brought tangible professionalization of postgraduate medical education in Bhutan at a very early stage of the Khesar Gyalpo University of Medical Sciences of Bhutan. The quantitative and qualitative data supported the overall positive impact it has had on the professional development of the teacher, improvement in their self-efficacy and the improvement in their competency to deliver the postgraduate curriculum. There was a significant improvement in the domains around the ability to teach relevant subject matters and developing creative ways to cope with system constraints. Assessment of self-efficacy was important at this stage because most of our faculty members are clinicians at the hospital and teaching was a new job responsibility [2, 15, 21]. The improvement in the scores of teacher self-efficacy and competency are promising results of a structured FDP in an environment where the systems in the University is taking its maturation steps.

The qualitative interviews demonstrated the importance and relevance of FDPs in making the paradigm shift in medical education and in equipping them with skills to deliver the teaching of the six core competencies of the Accreditation Council for Graduate Medical Education [9]. With the introduction of the revised curriculum for residency training in 11 departments, the FDPs have helped the teachers make the transition from conventional forms of teaching and assessment to workplace-based environment [1, 8, 12]. These workshops have already resulted in the improvement of teaching competency score and brought about changes in the methods of teaching such as the adoption of online means, and use of objective tools in bed-side teaching or in teaching procedures [12, 20].

While about three-quarters of students in the University in 2015 reported a positive impression on the use of PowerPoint presentations [22], the FGD recorded mixed views amongst faculty members regarding its use. The quantitative analysis did not show the change in the attitude of the faculty members regarding PowerPoint presentation. The faculty members favoured bedside teaching and hence the importance of workplace-based learning and assessment. Case-based discussions and direct observation of history taking, physical examination and procedural skills have benefitted in the organization of subject matter and objective assessment of both knowledge and skills and legitimizing trustable professional activities under the Bhutan Medical and Health Council regulations [9, 12]. The FDPs also have brought changes such as the introduction of OSPEs in the postgraduate entrance exam at the Faculty of Postgraduate Medicine [12]. Other workplace-based assessment methods such as 360 degrees assessment are aimed at the development of the affective domain of the student.

The FDPs have resulted in the development of quality check in the residency programme and in the professional development of the teachers. The departments have taken context-specific measures to create an effective learning environment for their residents such as the development of lecture halls, skills lab and introduction of inter-departmental conferences. However, there is a resounding call for more support needed for FDPs and the residency programme from the hospital administrators. Currently, the hospital is an autonomous organization that is partly controlled by the Ministry of Health while the University is an independent organization under an act of Parliament. There is an urgent need for harmonization of these institutions to provide more support to the residency and the faculty development programmes. Despite these challenges, the faculty members scored a significant improvement in the self-efficacy theme around developing creative ways to cope with the constraints in the system (Table 1) and shared several examples of creating better avenues for their residents to learn.

In 2014, the implementation of residency and the faculty development programmes were received with doubts, concerns, and insecurities of sailing through uncharted waters. Such reactions are common barriers to the introduction of professional tenets in medical education in a setting where conventional means were already established [19]. The Faculty of Postgraduate Medicine had an advantage of inducting FDPs while the first batch of residents was still undergoing training. However, not all of the current teaching faculty members have been trained in FDPs. In addition, to sustain these changes, there is a need for a dedicated medical education centre in the university to provide training to new faculty members and refresher courses to those trained.

The FDPs demand the teachers to commit to the continuous development of their knowledge and skills in medical education and apply them in their respective settings [23]. To bring about the desired outcome of FDP in residency training, the FDPs must be allowed to mature in the context and challenges specific to our setting. The current evaluation of impact aimed at assessment of the learner’s reaction, modification of their attitudes/perceptions, and acquisition of their knowledge/skills [23]. The interviews reported some changes in the practices at the level of the departments and at Faculty of Postgraduate Medicine. Long-term benefits to residents, specific benefits to patients and communities, and benefits to the teachers themselves need to be studied.

The FDPs have not only benefitted resident learning and improvement in service delivery at the hospital, but it has also brought improvement in the working environment amongst the faculty members through better communication skills. An increased proportion of faculty members reported the positive impact of FDPs on their communication skills with regard to delivery of subject knowledge and effective communication to bring about behavioural and attitudinal change in their students (Table 2). The FGD brought examples of the benefits of better communication skills for both residency training and patient care.

The current approach to faculty development at the Faculty of Postgraduate Medicine has been generic and directed at introduction and adoption of the principles of medical education. Medical education is a fast-changing field with sub-specialization into specific fields of medicine and nursing [24–26]. In our context, the University needs a central medical education centre that will facilitate faculty development specific to the needs of the different programmes in its three Faculties of Postgraduate Medicine, Nursing and Public Health and Traditional Medicine. Such a central platform will also provide an avenue for inter-professional faculty development.

### Limitations and recommendations

Only the basics of medical education and assessment methods that were of urgent need were prioritized in this cycle of FDP. We did not compare the effectiveness of one type of FDP over another for a newly established medical university.

## Conclusions

The FDPs have brought increased self-efficiency and teaching competency among those who were trained. FDPs also brought tangible professionalization of postgraduate medical education. The challenges are the lack of a medical education centre to sustain the gains made through the past FDPs.

## Supplementary information

**Additional file 1.** Interview guide FGD.

## Data Availability

The datasets generated and/or analysed during the current study are available from the corresponding author upon request.

## Abbreviations

ACLS: Advance Cardiac Life Support
BLS: Basic Life Support
COREQ: Consolidated Criteria for Reporting Qualitative Research
DOPS: Direct Observation of Procedural Skills
FDP: Faculty Development Programme
FGD: Focussed Group Discussion
JDWNR Hospital: Jigme Dorji Wangchuck National Referral Hospital
OSCE: Objective Structured Clinical Examination
