# Supplementary material for: Impact of faculty development programme on self-efficacy, competency and attitude towards medical education in Bhutan: a mixed-methods study
Source: BMC Med Educ. 2019 Dec 21;19:468. doi: 10.1186/s12909-019-1904-4 (PMC6925861; doi:10.1186/s12909-019-1904-4)
Supplement: Supplementary file 1 — Additional file 1. Interview guide FGD. [file 12909_2019_1904_MOESM1_ESM.docx]

**Impact of Faculty Development Programme on Self-efficacy, Competency and Attitude towards Medical Education in Bhutan: a mixed-methods study**

**For teaching faculty members at Faculty of Postgraduate Medicine, Khesar Gyalpo University of Medical Sciences of Bhutan, Thimphu**

1. There are seven areas of teaching competencies that we assessed in the questionnaire: (a) communication skills (b) engaging and supporting all students (c) creating and maintaining effective environment (d) understanding and organizing subject matter (e) planning instruction and designing learning experiences (f) assessing student learning and (g) developing as a professional educator. **In your opinion, in which domain has this FDP brought about a major transformation in you?**
2. What are your experiences in the use of various teaching methods? Which ones do you use frequently?
3. What are your experiences in using various media of teaching? Which ones do you use frequently?
4. What are your experiences regarding the use of various assessment methods? How do you think it can deliver the new PG curriculum in KGUMSB?
5. What are the challenges that you face in adopting these teaching and assessment methods?
6. Five years down the line, where do you want to see the university’s FDP?
